# The sociolinguistic problems of English medium instruction in the Middle East and North Africa: Implications for epistemic access

**DOI:** 10.3389/fpsyg.2023.1084626

**Published:** 2023-02-09

**Authors:** Berrington Xolani Siphosakhe Ntombela

**Affiliations:** Department of Languages, School of Languages and Communication Studies, Faculty of Humanities, University of Limpopo, Sovenga, South Africa

**Keywords:** English medium instruction, globalization, sociolinguistic problems, internationalization, critical approach, epistemic access

## Abstract

The global spread and dominance of English in higher education has reached alarming heights. While there has been a drive to argue for the relevance and importance of education through local languages, English has snatched the biggest slice of the cake by subtly imposing itself as the sole global language of education. This paper interrogates the sociolinguistic problems posed by the hegemony of English language. It argues that globalization and internationalization work in tandem with neo-colonial and neoliberal operations to create a class of global citizens that must support the economic aspirations of English imperial expansion and sustenance. The arguments are drawn from the experiences of Middle East and North Africa as well as lessons from Eastern and Southern Africa. The paper adopts a critical approach in order to place urgency against the onslaught of English medium of instruction in global higher education. This is done by problematizing the rhetoric of globalized and internationalized education. The paper then draws conclusions on epistemic access in the context of burgeoning knowledge economies. It argues that English medium of instruction is implicated in stalling access to knowledge for the great majority in order to cater for and safeguard the economic dominance of the elite minority.

## 1. Introduction

English has spread across the globe to dominate the education sector. Although this has threatened the existence of local languages, “[m] uch of the literature in English as a “global” or “international” language has tended to be celebratory” (Skutnabb-Kangas and Phillipson, 2010, p. 80). For example, Oraif and Alrashed (2022) report a positive attitude on the use of English medium instruction, which replaced Arabic in a Saudi Arabian university. Furthermore, Knight (2013) argues that the demand for English medium instruction programs is based on the prestige of using English to access latest developments. Nevertheless, the global and international imperatives of the English language as a medium of instruction have sociolinguistic problems that can be analyzed through a framework of a critical approach.

Every human being is endowed with language not only as a means of communication, but as a means of interacting with the world. Although animals also have means of communication, human beings differ in the level of sophistication and ability to talk about hypothetical situations (Aitchison, 2008). What further distinguishes human beings from animals is their ability to acquire or learn any language they are exposed to naturally or formally in an education situation.

Because of this ability to learn languages in an education situation, languages are able to spread to far away territories. This makes other languages that can travel far dominate those that are confined to their territories. The current spread and dominance of English language in the world started from the imperial expansion of the British Empire where colonies were established across the length and breadth of the globe. In every colony, English language was imposed as the language of education, governance, commerce and the judiciary, to name a few.

Although colonialism might be said to have ended, the operations of globalization have a lot in common with colonialism (Skutnabb-Kangas and Phillipson, 2010). For example, during the colonial period, dependence was placed on the colonizer for educational, commercial, civil, and judicial transactions; in globalization, there is a similar dependence in that in order to function educationally, commercially, judicially etc. one must use the language of the former colonizer (Prah, 2018). This current dominant language for such transactions happens to be the English language.

This article argues that in the educational context, the dominance of English medium instruction has sociolinguistic problems which work against the success of many students and ordinary citizens. The Middle East and North Africa are used to demonstrate these sociolinguistic problems. In addition, the experiences of a few countries in Eastern and Southern Africa are used to cautionary predict the future direction if the adoption of English medium instruction is not problematized.

The article has adopted a critical approach (Scott and Usher, 2004) in order to interrogate and critique the hegemonic dominance of English language and the adoption of English medium instruction in the Middle East and North Africa. Critical approach places urgency in the hands of the subordinate so that they are not helplessly bound to the status quo (Zink, 2021). In other words, the rationale for adopting a critical approach lies in its emancipatory end (Ntombela, 2021). In the context of this article, the critical approach is used to interrogate the ramifications of EMI in MENA.

## 2. Emi in MENA

In the Middle East, the establishment of the Gulf Cooperation Council (GCC) was influenced by the United States of America (Hanieh, 2011). There are six countries that make up the GCC: the United Arab Emirates (UAE), Kingdom of Saudi Arabia (KSA), Qatar, Sultanate of Oman, Kuwait, and Bahrain. All these countries have Arabic as a common language. Oil reserves in the rich GCC countries is one of the socio-economic similarities. Demographically, the population of the citizens of each of the GCC countries is outnumbered by the expatriate population.

Other similarities include reasons why each of the GCC countries adopted EMI. For instance, Saudi Arabia sought to move from oil-based economy to knowledge-based economy. She also sought to align education with the job market. In the process, English was adopted as the language of global academic capital (Barnawi, 2018). Nevertheless, the impact of the adoption of EMI to Arabic and its speakers was never considered.

The UAE also sought to align education with the job market. She also sought to transform the economy into a knowledge-based economy. With all these changes in the UAE, English has become the currency through which participation in the economy and education is realized (Park, 2016). For instance, teachers of English, and of maths and science in the UAE public schools are required to provide an IELTS score of 6.6 and 5.5, respectively, every two years in order to continue working (Barnawi, 2018). Similarly, the established utility of Arabic in the teaching of maths and science was never entertained.

Qatar also sought to align education with the job market. Like the UAE and KSA, Qatar sought to transition from an oil-based to a knowledge-based economy. This alignment entailed changing the education policy that adopted EMI whilst relegating Islam into oblivion (Abdel-Monein, 2016; Barnawi, 2018). English in Qatar is seen as a symbol and embodiment of modernity, economy, entrepreneurship and globalization (Barnawi, 2018). In the same vein, Qatar did not interrogate the repercussions of EMI especially given the viability of Arabic in accomplishing the educational ends that have been shifted to English.

The Sultanate of Oman also sought to harmonize education with the demands of the job market. Furthermore, the harmonization of the education system was meant to increase the participation of Omani citizens in the knowledge-based economy. Similarly, in realizing these developments, Oman adopted English which has since dominated aspects of society including, business, education, law, and communication (Tekin, 2016; Barnawi, 2018). In the process of adopting EMI, Oman failed to realize the resultant disharmony between the sociolinguistic aspects and the job market.

Kuwait as well sought to respond to the mismatch between education and the job market. Similarly, Kuwait sought to transition into a knowledge-based economy and decrease dependence on oil-based economy. English has therefore been adopted as a means of accessing the global knowledge economy. Nonetheless, Kuwait might seem to have adopted a fallacious belief that access to global knowledge can only be achieved when English is a medium of instruction (Brock-Utne, 2017).

In the same vein, Bahraini’s economic challenges made it imperative to transition into a knowledge-based economy. In the same way, Bahrain had to make sure that education is aligned to the job market. All this have been addressed by adopting EMI policy, pedagogy and practice (Barnawi, 2018). Similarly, Bahrain does not seem to have considered the sociolinguistic problems of EMI especially among the majority users of Arabic.

Countries in North Africa are in different stages of adopting EMI for various reasons. For instance, Morocco, which is francophone, has French as the medium of instruction, and Arabic as the official language. However, the attitudes are warming up toward EMI because of French’s association with colonialism. But most importantly, English is associated with modernity, globalization and prospects of ushering economic participation in the global market (Troudi, 2022). Again, Morocco seems to be jumping from one foreign language to the other, sidelining Arabic as the most viable language.

Tunisia’s situation is not very different from the Moroccan’s. French, inherited from the colonial system, has a strong hold in the education system, and Arabic is the official language. However, the benefits of the French education have largely gone to the elite. French is dominant in higher education and Arabic features mostly in primary and secondary education. There is a growing appetite for EMI which is associated with economic development, globalization, and modernization (Troudi, 2022). However, Tunisia does not seem to have problematized the confinement of Arabic to primary and secondary education whilst promoting French and English in higher education.

Algeria is also francophone. There is a continuing debate between those who adhere to French in education and those who advocate for Arabic as a symbol of religious identity. However, French is reported to be declining and English is increasingly “seen as a solution to the educational, technological and economic problems of the country” (Troudi, 2022, p. 137). Nonetheless, it remains to be seen how English could solve problems that French could not solve.

In Libya, Arabic is the dominant language in education. The brief Italian colonization did not have the impact France had in Morocco, Tunisia and Algeria. Nevertheless, a few private institutions of higher learning offer tuition in the medium of English. Those attending such institutions favor being taught in English even though it poses challenges to them academically. English is being associated with global communication and access to the job market locally and internationally (Troudi, 2022). Clearly, it is anomalous that English is embraced despite the academic challenges it poses.

EMI is more established in Egypt because of the British colonial influence. Although Arabic remains a practical language throughout the schooling years, higher education is being dominated by EMI. Consequently, Egypt has established experimental language schools with an aim of preparing learners for smooth transition into EMI in higher education. This is all because English is seen as a solution to the country’s “objectives of modernization through more vital global contribution, economic and business development, and technical and scientific research” (Troudi, 2022, p. 143). Paradoxically, the practicalities of Arabic are able to effectively achieve the country’s objectives.

## 3. Lessons from Southern and Eastern Africa

The major languages spoken by millions as a mother tongue that make Southern and Eastern Africa dynamic include Amharic, Chichewa, isiZulu, Kikuyu, Kinyarwanda, Kirundi, Luganda, Malagasy, Oromo, Somali, and Swahili (Trudell, 2016). However, colonialism brought European languages that continue to dominate the linguistic landscape of Eastern and Southern Africa. These include English, French and Portuguese. Of these three, English is the most dominant and the ever expanding.

Mozambique, for example, has Portuguese as the medium of instruction throughout education and as an official language. Portuguese was inherited from a colonial period, and during the time of independence, it was spoken by only 7% of Mozambicans. English was introduced as a subject in public schools and its dominance continues to grow (Mendonca, 2014). Research, however, has indicated that students have done much better when schooled in their mother tongue (Trudell, 2016) while English proficiency remains a challenge for graduates at the workplace. In fact, the addition of English to Portuguese is not expected to help the population navigate the educational space.

Namibia was first colonized by Germany, but later, South Africa took the administration of the colony. For the period that South Africa was the administrator, Afrikaans was the dominant language. When Namibia gained independence from South African administration, English was adopted as a medium of instruction from Grade 4. However, some schools have adopted English as a medium of instruction from lower grades, which has created frictions about the promotion of indigenous languages that are meant to be used as media of instruction up to Grade 3 (Totemeyer, 2010; Trudell, 2016). Nevertheless, the use of EMI has not been helpful in giving learners access to content knowledge and there is a feeling by many educationists that the policy that advocates for the use of EMI is failing students (Harris, 2011; Trudell, 2016).

Burundi is a former Belgian colony and therefore francophone. However, Kirundi is listed as the national and official language. In education, Kirundi is used as the medium of instruction in primary school with French taking over from Grade 5 upwards. In a Kirundi monolingual country, the use of French as a medium of instruction is problematic. However, the elites believe that education in an African language will lower the standards and, therefore, advocate for French as an academic and professional language (Rwantabagu, 2014; Trudell, 2016). In addition to Kirundi and French, English and Swahili are learnt from Grade 1 even though less than 5% of the population speaks, English and Swahili. There does not seem to be any pedagogical justification for adding these languages which have only added to the pupils’ learning load.

## 4. Globalization and internationalization

The rhetoric surrounding globalization is often punctuated with the role of English as a *lingua franca* in communication, business, entertainment, science and technology, and so forth. This is because the world is conceived to have collapsed into a global village with English playing a major role. The place of English is cemented by the fact that the movement of global citizens converges into the English-speaking territory, the West. Because these citizens hail from various linguistic backgrounds, they require a common language that is utilized in the host country’s main transactions. The result is that since English presides over all aspects of life, that is, commerce, education, entertainment, science, and technology, and so forth; it becomes the default language in the global village. The interlocutors, therefore, bring with them English expressions that are influenced by languages of the countries of their origin. As a result, English *lingua franca* is characterized by a plethora of Englishes’ (Bolton, 2004; Shin and Kubota, 2008; Kachru, 2011; McKay, 2011).

Internationalization in the context of higher education entails offering tuition in the medium of English, which is considered an international language (Crystal, 2003). The English in higher education programs is prestigious in comparison to *lingua franca* English. The main reason is that the standardized form of English is envisaged and expected in high stakes assessment right up to academic publications. It is very common to have native speakers of English placed as a reference in such cases. In fact, the academic literacy programs in many institutions are dominated by English language proficiency and the default clients are non-native speakers of English.

## 5. Epistemic access

Language is essential in accessing knowledge. Epistemologically, knowledge relates to how reality can be described (Scott and Morrison, 2007). Epistemology is distinguished from ontology, which has to do with the nature of reality (Molinari, 2022). This means as every human being is endowed with language ability, access to knowledge is equally open to every humanity. Language in this case is an epistemological means and not ontology. To distinguish language epistemology from ontology; ontologically, language relates to a phenomenon common to all humanity as distinguished from animals. In other words, animals have a means of communication but only human beings have a language (Aitchison, 2008). However, language as a human phenomenon can be further distinguished from a particular language that distinguishes one human being (or a group of human beings) from another. For example, English, French, and Arab, as a people, can be distinguished from one another by English language, French language and Arabic language, respectively. Therefore, each human being can access language (ontologically) through a particular language (epistemologically).

Put differently, a particular language is what gives access to reality (epistemology), but that particular language is not reality itself (ontology). That is, English language, as an example of a particular language, is a means of accessing knowledge but English language itself is not knowledge. It is important to maintain this distinction. This means any particular language is endowed with equal means of accessing knowledge. However, when a particular language is ontologically conceived, it no longer becomes a means to an end but is itself an end.

## 6. Discussion

The current dominance of English language globally and in the Middle East and North Africa in particular is reminiscent of the dominance of other languages in history. A few examples include Aramaic, Greek, Arabic, Mayan, Manding, and Latin which were languages that spread in Eastern Mediterranean, Middle East and North Africa, Central America, West Africa, and Western Europe, respectively, through political and military conquest (Spolsky, 1998). The current spread of English language can be traced to colonialism which is currently represented by globalization.

The historical spread of the languages cited as examples destabilized other languages and people who spoke those languages. People lost their languages, cultures, identities and were assimilated into a singular cultural system. A lot of knowledge was also lost alongside languages, cultures and identities.

In the present situation, English language is taking over Arabic in MENA. In higher education, knowledge accessed through the English language is prioritized and legitimated. This means that knowledge accessed through Arabic is trivialized and marginalized. In fact, the Arabic language is conceived as a terrorist language that must be eradicated. For example, in Saudi Arabia, the aftermath of 9/11 saw the change of educational policies that elevated the EMI replacing the Arabic Medium Instruction (AMI). This was because the Arabic language was labelled as propagating Islamic fundamentalism that opposed the modernist ideals of democracy and tolerance as encapsulated in the English language (Barnawi, 2018). In a similar way, the EMI replaced the AMI in Qatar and was praised in the West as though such a replacement meant replacing Islam with English (Karmani, 2005). English in that way was not just conceived as a neutral language but a religious system that represented the modernist ideals of democracy, tolerance and capitalism.

This shows that EMI does not seem to represent a sound pedagogical solution than a linguistic conquest. Unfortunately, such conquest has negative repercussions for ordinary students who could be better served by accessing knowledge through their languages. The adoption of EMI is usually justified on the grounds that English presides over every knowledge, be it educational, technological, economic or scientific. The extension of such a justification is that a person is only taken to have educational, technological, economic and scientific knowledge only if it is accessed and expressed through the English language. This results from conflating epistemology with ontology. That is, knowing English is taken as having knowledge and not just one of the many ways of accessing knowledge. For instance, mathematical, economic, technological or educational concepts can be accessed through Arabic the same way they can be accessed through the English language – i.e., mathematical, economic, technological or educational concepts, for example, are not necessarily English concepts and need not be accessed only through the English language. In fact, these concepts would be better accessed through the language understood by the learners. Otherwise, as it is the case for the majority of learners, access to knowledge is jeopardized.

The examples from Southern and Eastern Africa show that when a language that is not understood by learners is made to be the medium of instruction, learning is slowed down and success evades learners. This reality is already experienced by the majority of students in the Middle East and North Africa who are not receiving education through the language they understand.

Whilst a few, usually members of the minority elite, benefit from EMI, the majority are left without access to the benefits of better jobs, better economic participation and better education, that are promised to be offered by EMI. This situation perpetuates a cycle of inadequacy and dependence where EMI is in turn presented as a solution.

## 7. Conclusion

The dominance of English language in higher education has not produced the often-promised results of access to global knowledge and participation in the global economy. Instead, the majority of students are denied access to knowledge and cannot participate in the economy locally and internationally. This is a sociolinguistic anomaly because they have their own languages that are equipped with all capabilities of accessing and expressing knowledge. Until this situation is justly addressed, the gap between the minority that has access to the English language and the majority that does not have such access will continue to widen. The simplest solution is to use the Arabic language to access educational content knowledge and still learn English as a subject for international communication.

## Data availability statement

The raw data supporting the conclusions of this article will be made available by the authors, without undue reservation.

## Author contributions

The author confirms being the sole contributor of this work and has approved it for publication.

## Conflict of interest

The author declares that the research was conducted in the absence of any commercial or financial relationships that could be construed as a potential conflict of interest.

## Publisher’s note

All claims expressed in this article are solely those of the authors and do not necessarily represent those of their affiliated organizations, or those of the publisher, the editors and the reviewers. Any product that may be evaluated in this article, or claim that may be made by its manufacturer, is not guaranteed or endorsed by the publisher.
